# Obstetric near-miss and maternal mortality in maternity university hospital, Damascus, Syria: a retrospective study

**DOI:** 10.1186/1471-2393-10-65

**Published:** 2010-10-19

**Authors:** Yara Almerie, Muhammad Q Almerie, Hosam E Matar, Yasser Shahrour, Ahmad Abo Al Chamat, Asmaa Abdulsalam

**Affiliations:** 1 Department of Obstetrics & Gynaecology, Faculty of Medicine, Damascus University, Damascus, Syria; 2 Diana, Princess of Wales Hospital, Scartho Road, Grimsby DN33 2BA, UK. Previously: Medical Student, Faculty of Medicine, Damascus University, Damascus, Syria; 3 North Middlesex University Hospital, Sterling Way, London N18 1QX, UK. Previously: Medical Student, Faculty of Medicine, Damascus University, Damascus, Syria

## Abstract

**Background:**

Investigating severe maternal morbidity (near-miss) is a newly recognised tool that identifies women at highest risk of maternal death and helps allocate resources especially in low income countries. This study aims to i. document the frequency and nature of maternal near-miss at hospital level in Damascus, Capital of Syria, ii. evaluate the level of care at maternal life-saving emergency services by comparatively analysing near-misses and maternal mortalities.

**Methods:**

Retrospective facility-based review of cases of near-miss and maternal mortality that took place in the years 2006-2007 at Damascus Maternity University Hospital, Syria. Near-miss cases were defined based on disease-specific criteria (Filippi 2005) including: haemorrhage, hypertensive disorders in pregnancy, dystocia, infection and anaemia. Main outcomes included maternal mortality ratio (MMR), maternal near miss ratio (MNMR), mortality indices and proportion of near-miss cases and mortality cases to hospital admissions.

**Results:**

There were 28 025 deliveries, 15 maternal deaths and 901 near-miss cases. The study showed a MNMR of 32.9/1000 live births, a MMR of 54.8/100 000 live births and a relatively low mortality index of 1.7%. Hypertensive disorders (52%) and haemorrhage (34%) were the top causes of near-misses. Late pregnancy haemorrhage was the leading cause of maternal mortality (60%) while sepsis had the highest mortality index (7.4%). Most cases (93%) were referred in critical conditions from other facilities; namely traditional birth attendants homes (67%), primary (5%) and secondary (10%) healthcare unites and private practices (11%). 26% of near-miss cases were admitted to Intensive Care Unit (ICU).

**Conclusion:**

Near-miss analyses provide valuable information on obstetric care. The study highlights the need to improve antenatal care which would help early identification of high risk pregnancies. It also emphasises the importance of both: developing protocols to prevent/manage post-partum haemorrhage and training health care professionals to manage infrequent but fatal conditions like sepsis. An urgent review of the referral system and the emergency obstetric care in Syria is highly recommended.

## Background

Since the declaration of the Millennium Development Goals (MDGs) in 2000, reproductive health, the 5th goal of the MDGs, has been recognised as fundamental for human development. National governments and the international community have increasingly embraced language which supports reproductive health. However, re-orienting policies and programmes has been more challenging, particularly in the Middle East and North Africa region (MENA) [1]. Decision-makers here are still facing major difficulties in allocating the limited available resources. A major contributing factor is the lack of accurate data that identifies which women are at highest risk of maternal death and what actions will best reduce that risk [2].

Each year approximately 529 000 women die of complications during pregnancy and delivery; almost all of which (99%) occur in low-resource countries [3]. However, despite the high maternal mortality ratios in many of the centres in resource-poor settings, maternal deaths are rare in absolute numbers per centre. This does not allow detailed quantification of the associated risk factors and determinants that are locally important.

Near-miss is a serious adverse event that leads to harm and morbidity in the mother, but from which she survives [4]. Since 'near-miss' is somewhat more frequent than maternal deaths, interest has grown in using it as an indicator of the quality of obstetric care [5]. Because surviving a near-miss occurs mainly because of the care provided, reviewing near-misses has the potential of highlighting deficiencies and positive elements in the obstetric care of any health system [6].

Essentially, three different methods have been used for identifying near-miss cases. These different approaches depend on either a set of clinical criteria defining common diagnostic categories; a set of management-based criteria related to specific interventions or on organ(s)/system(s) failure [5].

Although a considerable body of obstetric near-miss analyses have been recently conducted in developing countries [7-11], such evidence is scarce in the MENA region. Syria is a lower-middle income country in the MENA region [12]. Its national health expenditure accounts for 5.9% of the Gross Domestic Product (GDP) which equals only 52 $ per capita (in the UK, it is 16.5% of the GDP which equals 2,434 $ per capita) [12]. Almost 70% of deliveries are facility-based [13]. According to national figures, the MMR has declined from 143 per 100 000 live births in 1990, to 58 per 100 000 in 2005 [14,15]. UN estimate, however, shows higher figures (180 per 100 000 in 1990 and 130 per 100 000 in 2005) [16].

In this retrospective study, we aim to document the frequency and nature of maternal near-misses at a hospital level in Damascus, the Capital of Syria. We also aim to evaluate the level of care at the maternal emergency services in Syria by comparatively analysing maternal near-misses and mortalities. This review is expected to serve as a guiding tool for policy-makers by highlighting the most common and the infrequent but serious maternal morbidities to help direct the expenditure of the modest national health budget towards health care priorities.

## Methods

### Settings

We conducted a facility-based retrospective study between February and July 2008 for all admissions to Damascus Maternity University Hospital (DMUH), the women teaching hospital for Damascus University, in the years 2006 and 2007. DMUH is one of two maternity referral hospitals in Damascus which serve a region populated by over 3 million inhabitants. This 250-bed hospital has approximately 21,000 admissions yearly, 60% of which are accepted in labour and pregnancy units. This study was approved by Damascus Faculty of Medicine (21 Jan 2007).

### Definition of cases

A near-miss event is 'a woman who nearly died but survived a complication that occurred during pregnancy, childbirth or within 42 days of termination of pregnancy' [17].

The disease-specific criteria set by Filippi *et al *[18] were employed to identify near-miss cases. This set of criteria is based on five main diagnostic domains: i. severe haemorrhage (leading to shock, emergency hysterectomy, coagulation defects and/or blood transfusion of ≥ 2 litres); ii. hypertensive disorders in pregnancy which include eclampsia, severe pre-eclampsia (BP >140/90 mmHg and proteinuria > 1g/24hrs) or HELLP syndrome; iii. sepsis defined as a temperature < 36°C or > 38°C and clinical signs of shock (systolic BP < 90 mmHg and heart rate > 120 beats per minute); iv. dystocia which includes uterine rupture and impending rupture; v. severe anaemia (haemoglobin < 6 g/dl) or clinical signs of severe anaemia in women without severe haemorrhage.

In 2009, the World Health Organisation (WHO) published a consensus on maternal near-miss definition and set a criteria for cases identification [17]. These criteria, which are based on dysfunctional organs, were not used by our study as the study commenced before the publication of the new WHO consensus.

All maternal near-misses which occurred before or after arrival at the hospital were included in this study. These were further classified into direct (resulting from complications occurring during or after pregnancy) or indirect (resulting from previously existing disease aggravated by pregnancy). Causes of maternal mortality, as defined by the International Classification of Diseases (ICD-10)[19], were also reported.

### Data collection and analysis

Maternal deaths and near-miss cases were retrospectively identified by searching the Medical Records Register at DMUH. Each patient's discharge summary includes 'primary' and 'secondary' diagnoses sections. It also has a 'complication' section, where all complications which happened as a result of either the primary or the secondary diagnoses are listed. Discharge summaries also include 'investigations' (like ultrasound, echocardiogram, etc) and 'interventions' (like hysterectomy, blood transfusion, etc) sections.

Using the data documented in the admission/discharge sheets of the hospital, we identified cases where any of Filippi's main disease-specific categories was mentioned (potential inclusion). The full notes of these cases were then scrutinised and carefully checked against Filippi's criteria for final inclusion. The data collection was done by 3 investigators using a pre-coded form specially developed for this study. All data was then inserted into a Microsoft Excel spreadsheet. Causes of maternal deaths were generally based on clinical or surgical findings. Data on deliveries and live births were derived from statistics reported by the DMUH's Statistical Department.

For each case, we collected data on demographic characteristics including patient's age, parity, previous deliveries, and gestational age at delivery. We also collected data on the nature of the obstetric complication(s) responsible and where it developed (home, private doctor, maternity centre, and hospital), type of delivery, foetal outcomes, any ICU admissions including length of stay, and any special procedure carried out during the care of the woman. Special procedures were defined as propaedeutic or therapeutic interventions not normally used during prenatal, intra-partum, or postnatal care e.g. echocardiography, hysterectomy, transfusion ≥ 4 units, electric cardioversion and iliac arteries ligation.

SPSS 13.0 software (SPSS Inc, IL, USA) was used for descriptive statistical analysis. Results are presented as frequencies, percentages and descriptive statistics. MMRs and MNMRs were calculated using live births as the denominator. In order to evaluate the standard of care provided for each disease category, we calculated the mortality index for each obstetric condition. This was defined as the number of maternal deaths due to a particular obstetric condition divided by the sum of near-miss morbidities and maternal deaths which resulted from this condition, expressed as a percentage [20].

## Results

During the 2-year period reviewed, there were 28 025 hospital deliveries, 27 350 live births, 901 near-miss cases and 15 maternal deaths. This resulted in a total MMR of 54.8/100 000 live births. The total MNMR was 32.9/1000 live births and the total mortality index for near-miss cases was 1.67% (near-miss/fatality ratio 60:1). Most of the cases (n = 839, 93%) were referred to the hospital with the complication while only 7% developed the complication within the hospital.

During the study period, 54% of women with near-miss delivered by caesarean section (rate is 20% in all hospital deliveries). Foetal outcomes were 82% liveborn; 11% abortions and 6% stillbirths. Other demographic characteristics of women who sustained near-miss complications and those who died are presented in table 1.

**Table 1:** Characteristics of near miss/maternal deaths at Damascus Maternity University Hospital, Syria

Characteristics	Near miss n = 901(%)	Maternal death n = 15(%)
**Age μ **± **SD yrs**	28.4 ± 7.1	29.1 ± 7.1
**Parity**: Median (range)	2 (0-15)	2 (0-8)
0	252 (28.0)	1 (6.7)
1-3	369 (40.8)	9 (60.0)
4 or more	280 (31.1)	5 (33.3)
**Previous liveborn babies**	2 (0-12)	2 (0-5)
**Previous stillbirth**	0 (0-8)	0 (0-3)

**Gestational age at delivery**		
GA < 28 wks	110 (12.2)	3 (20.0)
GA 28-32 wks	112 (12.4)	1 (6.7)
GA 33 -36 wks	176 (19.5)	1 (6.7)
GA >36 wks	503 (55.8)	10 (66.7)

**Complication development**		
During hospitalisation	62 (6.9)	6 (40.0)
Referred:	839 (93.2)	9 (60.0)
○ Home	604 (67.0)	3 (20.0)
○ Private doctor	99 (11.0)	1 (6.7)
○ Maternity centre	45 (5.0)	0 (0.0)
○ Hospital	87 (9.7)	4 (26.7)
○ Not specified	4 (0.4)	1 (6.7)

**Type of delivery**		
○ Vaginal	412 (45.7)	5 (33.3)
○ Caesarean section*	489 (54.3)	10 (66.7)

### Maternal deaths

14 (93.3%) of the 15 maternal deaths wer

e due to causes directly related to pregnancy, with severe late pregnancy haemorrhage leading the causes (n = 9), followed by hypertensive disorders (eclampsia, n = 2) and sepsis (n = 2). One additional death followed uterine rupture and one death was due to an exacerbation of a pre-existing cardiac problem. The mean 'stay in hospital to death' period was 2.6 days (SD = 4.2), table 2. Eight of the deceased women (53%) were admitted to ICU. Foetal outcomes were 11 liveborn, 2 abortions and 2 stillbirths.

**Table 2:** Classification of complications using maternal death and near miss morbidity upon and after arrival at Damascus Maternity University Hospital, Syria

Diagnosis		Near Miss (NM)				
				
	Mortality N = 15(%)	Total N (%)	In hospital n (%)	Referred n (%)	NM/1,000 live births	Mortality index (%)
Direct causes:						
**Hypertensive disorders**	**2 (13.3)**	**468 (51.9)**	**16 (25.8)**	**452 (53.9)**	**17.1**	**1:234 (0.4%)**
○ Severe pre-eclampsia	-	415 (46.1)	7 (11.3)	408 (48.6)		
○ Eclampsia	2 (13.3)	47 (5.2)	9 (14.5)	38 (4.5)		
○ HELLP syndrome	-	6 (0.7)	0 (0)	6 (0.7)		
						
**Severe Haemorrhage**	**9 (60.0)**	**310 (34.4)**	**32 (51.6)**	**278 (33.1)**	**11.3**	**1:34 (2.8%)**
*Early pregnancy*						
○ Ectopic pregnancy	-	43 (4.8)	0 (0)	43 (5.1)		
○ Abortion	-	14 (1.6)	0 (0)	14 (1.7)		
○ Hydatidiform mole	-	9 (1.0)	0 (0)	9 (1.1)		
*Late pregnancy*						
○ Placenta praevia	2 (13.3)	75 (8.3)	4 (6.5)	71 (8.5)		
○ Abruptio placentae	1 (6.7)	71 (7.9)	3 (4.8)	68 (8.1)		
○ Uterus atonia	6 (40.0)	83 (9.2)	21 (33.9)	62 (7.4)		
○ Other PPH	-	15 (1.7)	4 (6.5)	11 (1.3)		
						
**Sepsis/infection**	**2 (13.3)**	**25 (2.8)**	**2 (3.2)**	**23 (2.7)**	**0.9**	**1:12 (7.4%)**
○ Sepsis	1 (6.7)	12 (1.3)	0 (0)	12 (1.4)		
○ Amniotitis	1 (6.7)	12 (1.3)	2 (3.2)	10 (1.2)		
○ Septic abortion	-	1 (0.1)	0 (0)	1 (0.1)		
						
**Dystocia**	**1 (6.7)**	**34 (3.8)**	**6 (9.7)**	**28 (3.3)**	**1.2**	**1:34 (2.9%)**
○ Uterine rupture	1 (6.7)	23 (2.6)	6 (9.7)	17 (2.0)		
○ Impending rupture	-	11 (1.2)	0 (0)	11 (1.3)		
						
**Severe anaemia**	**0 (0)**	**30 (3.3)**	**2 (3.2)**	**28 (3.3)**	**1.1**	**N/A**

Indirect causes:						
**Previous medical condition**	**1 (6.7)**	**34 (3.8)**	**4 (6.4)**	**30 (3.6)**	**1.2**	**1:34 (2.9%)**
○ Cardiac	1 (6.7)	15 (1.7)	3 (4.8)	12 (1.4)		
○ Haematologic	-	15 (1.7)	1 (1.6)	14 (1.7)		
○ Others*	-	4 (0.4)	0 (0)	4 (0.5)		

**Total**	**15 (100)**	**901 (100)**	**62 (100)**	**839 (100)**	**32.9**	**1:60 (1.7%)**

*Epilepsy (n = 2), SLE (n = 2):

### Near-miss events

The disease profile for near-miss morbidity differed from that of maternal mortality (table 2). The most common types of near-miss cases fall under the diagnostic categories of severe hypertensive disorders (52%, MNMR = 17/1000 live births) or severe haemorrhage (34%, MNMR = 11/1000 live births). Most events due to severe haemorrhage developed in the later part of pregnancy (79%, n = 244) mainly due to uterus atonia (n = 83), placenta praevia (n = 75) or abruptio placentae (n = 71). The mortality indices for severe hypertensive disorders and severe haemorrhage were 0.4% and 2.8%, respectively. Sepsis (0.9/1000 live births) and dystocia (1.2/1000 live births) were uncommon causes of near-miss. However, they showed high-mortality indices of 7.4% and 2.9%, respectively (table 2).

The majority of the near-miss cases (93%) were referred in critical condition from other facilities namely traditional birth attendant homes (67%), primary (5%) and secondary (10%) healthcare units and private practices (11%) within Damascus city and countryside. This figure varied according to categories (97% in severe hypertensive disorders; 92% in sepsis, 90% in severe haemorrhage and 82% in dystocia). All severe haemorrhage in early pregnancy cases (n = 66) were referrals while 13% (n = 32) of late pregnancy haemorrhages developed in the hospital.

Two hundreds and forty five women with near-miss (27%) were admitted to ICU, with a mean stay of 3.5 days (table 3). 375 special procedures were carried out in 312 women which included 43 hysterectomy, 14 cardioversion, 15 echocardiography, 5 iliac arteries ligation and 187 transfusion of ≥4 units of blood products.

**Table 3:** Admission to ICU oby near-miss diagnosis

Near miss	N	Admission to ICU n (%)	Stay (days)
Severe haemorrhage	n = 310	107 (34.5)	4.3 ± 5.8
Hypertensive disorders	n = 468	96 (20.5)	2.9 ± 1.7
Sepsis	n = 25	5 (20.0)	3.2 ± 2.6
Dystocia	n = 34	17 (50.0)	3.3 ± 2.8
Severe anaemia	n = 30	5 (16.7)	4.6 ± 0.8
Medical condition	n = 34	15 (44.1)	4.0 ± 3.6

**Total (N = 901)**		245 (27.2)	3.5 ± 3.9

## Discussion

This is the first attempt to document both maternal mortality and severe morbidity in a Syrian setting. It shows a MNMR of 32.9/1000 live births, a MMR of 54.8/100 000 live births and a relatively low mortality index of 1.7%. Hypertensive disorders and haemorrhage were the leading causes of near-misses accounting for almost 86% of cases. Haemorrhage was the leading cause of maternal mortality (60%) while sepsis had the higher mortality index (7.4%). Most of the cases (93%) had near-miss upon arrival at the hospital. Almost quarter of near-miss cases were admitted to ICU.

The MNMR which this study describes (32.9 per 1000 live births) is located within the wide range of ratios reported in studies from other developing countries which used similar criteria for near-miss definition (12.3 - 82.3 per 1000 deliveries) [5]. On the other hand, the study describes a relatively low mortality index of approximately 1.7% (1:60), which indicates that for every 60 women who survived life-threatening complications in this centre, one maternal death was also recorded. This ratio, which reflects the overall standard of obstetric care, is slightly better than what studies from other developing countries reported (1:28 in Bolivia [7], 1:62 in Brazil [9], 1:15 in Benin [18], 1:18 in Cote d'Ivoire [18]) but still far from the 1:117-223 ratios reported in studies from Western Europe which used similar case definition criteria [21,22].

Studying cases of women who nearly died but survived a complication during pregnancy, childbirth or postpartum (maternal near-miss) is increasingly recognised as a useful means to examine the quality of obstetric care. Nevertheless, routine implementation and wider application of this concept in reviewing clinical care has been limited due to the lack of a standard definition and uniform case-identification criteria.

The WHO initiated a process aiming to develop a uniform set of criteria for identifying maternal near-miss cases. According to the new WHO criteria, published in 2009, near-miss cases are identified by dysfunctional system (cardiovascular, respiratory, renal, haematology, hepatic, neurologic) based on a set of clinical criteria, laboratory markers, or management-based proxies which were strictly defined [17]. It is hoped that this new consensus will facilitate reviews of near-miss to help monitoring and improving obstetric care.

In 2008, when the data collection of our study commenced, the WHO consensus on maternal near-miss definition was not yet published. The clinical criteria method, which we chose to define our near-miss cases, has many advantages including its easy interpretation, retrospective nature, and ability to assess both complication rates and quality of care of a particular disease [23].

Although case identification using admission to ICU is easier; it would have heavily underestimated severe morbidity, as generally <10% of near-miss cases in low-resource settings receive intensive care [11,24] (26.6% in our study). Besides, criteria for admission to ICU is variant and depends on the availability and capacity of ICU and on the institutional guidelines for ICU admission [25]. Organ system-based criteria, on the other hand, are regarded as the most specific means of identifying near-misses. However, this methods requires the ready availability of laboratory tests and medical technologies, which impedes its use in many low-resource local settings [6,25].

Similar to findings reported in many previous studies [7-9,11], hypertensive disorders and haemorrhage were the leading causes of near miss morbidities accounting for almost five-sixth of all cases.

As for hypertensive disorders, which make up almost half the maternal morbidities, 97% of cases developed upon arrival to the hospital. This exposes a weakness in early detecting pre-eclampsia; possibly due to poor antenatal care and follow-up. Hypertensive disorders, however, contributed to only 13% of maternal deaths (mortality index 240:1) which implies effective management of these complications upon arrival at hospital.

Of the various types of life-threatening obstetric haemorrhage, postpartum haemorrhage (PPH) constituted the greatest danger to affected women. While accounting for almost tenth of the maternal morbidities, PPH is responsible of 40% of maternal deaths. This suggests that either women had no access to care or poor care was provided by health professional [26].

Another area that needs improvement is management of sepsis which has the highest mortality index (12:1). This, indeed, constitutes a significant threat to the survival of affected patients although it is the least frequent cause of life-threatening obstetric conditions (0.9/1000 live births).

One critical defect this study helped expose is the delay in referrals to higher levels of care. Surprisingly, the majority of women with near-miss morbidity (93%) arrived at the hospital in critical condition having been referred from other hospitals, private practices or homes. This fact underscores the significance of pre-hospital barriers even in this setting with its availability of free maternal health care [18].

One possible barrier is related to transporting patients to hospital. A recent study on maternal mortality in Syria showed that in 31% of maternal deaths where deliveries took place at home, women died on the way to the hospital. Besides, in 18% of hospital deliveries, women died in another hospital as they were referred from hospitals which refused to admit them due to incapability of dealing with such cases. Small proportion of women were transported by ambulance (6%), while almost two thirds of them were brought by private cars or taxis [27].

Another obstacle to timely arrival are to be found in 'the first delay' [28], which is generally described as a failure of recognition that a serious complication is at hand, or a delayed decision to seek medical assistance on the part of the woman and others present--including family members and traditional birth attendants [28,29].

However, even if women arrive late, much can be done to save them and their babies, and a review into the care received in the hospital may lead to positive changes in the procedures and resources available for managing these complications [30]. This fact is particularly important in light of the recent reports which showed that up to 91% of maternal deaths in Syria are judged to be preventable, with main contributing factors being poor clinical skills and competency of the health care provider [27].

It is important to mention that our study has limitations. Its retrospective nature carries the possibility of underestimating near-miss cases due to poor documentation [23]. Other limitations are related to the disease-specified methods which we used in identifying near miss cases. Definition of conditions may not always be straightforward. For example, not all women with eclampsia nearly die and not all women with an ectopic pregnancy are critically ill. Besides, common direct causes of maternal mortality such as pulmonary embolus could be ignored. Although the use of mortality index is helpful as an indicator of the effectiveness of treatment in near-miss cases, we recommend doing a separate study that assesses the quality of care in near miss cases more thoroughly. Finally, this study was done in a public hospital, and we recommend that future studies cover also the private sector where a high percentage of deliveries take place.

## Conclusion

Near miss cases share many characteristics with maternal deaths and can directly inform about obstacles that had to be overcome after the onset of an acute complication, hence providing valuable information on obstetric care. This allows for corrective action to be taken on identified problems to reduce related mortality and long-term morbidity. Based on our findings, we recommend a number of actions to avert future maternal deaths: i. improving antenatal care to help early identification of high risk pregnancies including pre-eclampsia, ii. developing protocols to prevent/manage post-partum haemorrhage including raising the awareness about using active management during third stage, iii. training obstetric health professionals on managing infrequent but fatal conditions like sepsis, iv. urgently reviewing the referral system and emergency obstetric care in Syria.

## Competing interests

The authors declare that they have no competing interests.

## Authors' contributions

YA was responsible for the study design, together with AA, MQA and HEM. YA, AA and YS collected the data. MQA and HEM did the analysis and wrote the first draft in consultation with YA. AA revised the manuscript and all authors agreed on the final draft.

## Pre-publication history

The pre-publication history for this paper can be accessed here:

http://www.biomedcentral.com/1471-2393/10/65/prepub
